# Red Cell Distribution Width and Prediabetes in Adults in Northern Sudan: A Case–Control Study

**DOI:** 10.3390/hematolrep15040066

**Published:** 2023-11-20

**Authors:** Ahmed A. Hassan, Bashir E. Ahmed, Ishag Adam

**Affiliations:** 1 Faculty of Medicine, University of Khartoum, Khartoum 11115, Sudan; ahmed.ahmed@research.sunderland.ac.uk; 2 Rashid Hospital, Dubai P.O. Box 4545, United Arab Emirates; bemohamad@dha.gov.ae; 3 Department of Obstetrics and Gynecology, Unaizah College of Medicine and Medical Sciences, Qassim University, Unaizah 56219, Saudi Arabia

**Keywords:** diabetes mellitus, prediabetes, red cell distribution width, glycated hemoglobin, age, body mass index

## Abstract

Diabetes mellitus (DM) is a major public health issue worldwide. Red cell distribution width (RDW) has been reported to have predictive value in several diseases, including DM. Few data exist on the association between RDW and the prediabetic stage. Thus, the present study aimed to investigate the association between RDW and prediabetes in adults in Sudan. This case–control study was conducted in Northern Sudan in 2022. The cases (n = 107) were prediabetic patients categorized according to the level of glycated hemoglobin (HbA1c), which ranged from 5.7% to 6.4%, while the controls (n = 107) were healthy participants. A questionnaire was used to collect the data. Standard methods were used to measure the HbAIc level and RDW. Logistic regression analysis was performed. The median (interquartile range (IQR)) of the RDW was significantly higher in prediabetic patients than in the controls (14.5% [13.8–15.3%] vs. 14.1% [13.6–14.7%], *p* = 0.003). Sex, educational level, occupational status, marital status, cigarette smoking, alcohol consumption, family history of DM, and body mass index were not associated with prediabetes. In the multivariate-adjusted model, higher age and higher RDW were associated with prediabetes. A positive correlation was found between RDW and HbA1c levels (*r* = 0.19, *p* = 0.006). In conclusion, this study supports the use of RDW as a predictor of DM.

## 1. Introduction

Diabetes mellitus (DM; especially type 2, T2DM) is a major global public health problem [1]. DM can lead to several complications, such as diabetic nephropathy, diabetic neuropathy, diabetic retinopathy, and cardiovascular disease [2]. According to the International Diabetes Federation (IDF), prediabetes is a term used to describe people with impaired glucose tolerance and/or impaired fasting glucose; prediabetes indicates a higher risk of developing T2DM in the near future and its related complications [1]. The American Diabetes Association (ADA) defined prediabetes as glycated hemoglobin (HbA1c) levels ranging from 5.7% to 6.4% [3]. Compared to fasting blood glucose, HbA1c is commonly used since it is not influenced by a person’s last meal; therefore, it is more practical, especially in rural and remote areas [4]. The IDF reported that, in 2021, 541 million adults were prediabetic and were thus at a high risk of T2DM [5]. Prediabetic patients can develop several complications in the near future, and if no appropriate measures are taken, these complications, including coronary artery disease and diastolic heart failure, may develop even before the development of full-blown diabetes [6]. Various factors, such as male sex, older age, low level of education, family history of diabetes, alcohol consumption, and body mass index, were reported to be associated with prediabetes [7,8]. Various predictors of DM, such as complete blood count (CBC) parameters, including red cell distribution width (RDW), have been reported [9]. A CBC is a common investigation requested by healthcare professionals, and RDW is one of its parameters. RDW measures the quantitative variation in the size of circulating red blood cells (RBCs).

It is a marker of “anisocytosis”, which is the red cell’s size variation, and it is currently calculated automatically using a hematology analyzer by “simply dividing the standard deviation (SD) of the mean corpuscular volume (MCV) by the MCV and multiplying the quotient by 100 to yield a percentage value (according to this equation (RDW-SD)/(MCV) × 100)”. The RDW-SD is an actual measurement of the width of the red cell distribution curve in femtoliters (fl). RDW is usually expressed as the RDW coefficient of variation (RDW-CV), and its normal values range from 11% to 15% for adults [10]. RDW-CV is usually written as RDW. While high values of RDW are of high health concern, low values of RDW are of low health concern. More attention has been given to an increased RDW as a useful biomarker for several diseases, including DM [9,11], cardiovascular diseases [12], autoimmune diseases [13], and DM-related mortality [14]. For example, Wang et al. in their cohort study included 16,971 Chinese adults (9956 men and 7015 women) and found that an increased RDW was associated with a high risk of developing DM; therefore, they recommended using RDW as a predictor of diabetes mellitus [11]. While some studies reported a positive association between RDW and DM [9,11,15], others reported no association [15,16]; however, the reviewed studies have reported a positive association between RDW and prediabetes [9,11,17,18]. Early diagnosis and prompt intervention are important for controlling DM and its complications (morbidity and mortality), especially in its early stages. Identifying a cost-effective screening tool that can detect DM at an early stage, such as RDW, is crucial in preventing the development of DM and its complications. However, this newly emerging screening tool (RDW) for predicting DM needs more research in order to be approved and introduced in routine healthcare services, especially in limited-resource settings such as Sudan. IDF statistics show that approximately 90% of people with undiagnosed diabetes live in low- and middle-income countries (LMICs) [1] where RDW is a routinely available blood test. However, some factors that influence RDW were reported, such as poor dietary patterns [19], a lifestyle involving smoking [20], and physical inactivity [21].

DM increases the burden on existing fragile healthcare systems in African countries, and Sudan is not an exception [22,23,24,25]. For example, a recent systematic review and meta-analysis that included 157 articles, with a total population of 20,350, revealed high proportions of undiagnosed DM in different communities of African countries (the pooled prevalence of undiagnosed DM was 5.37% and ranged from 4.57% to 6.81%), and the study recommended policymakers to consider diagnostic strategies in order to improve screening for undiagnosed DM cases for effective care, aiming to achieve a substantial reduction in diabetes-related complications and mortality [25]. In Sudan, DM and its complications represent a major health problem in different regions [22,23,24]. Investigating a promising tool (i.e., RDW) to identify the group at high risk of DM at the early stages and taking all necessary preventive measures to combat DM and its complications are essential [11]. While most existing studies have focused more on the association between RDW and established DM and its complications [26,27,28,29,30], only a few studies have focused on the association between RDW and the prediabetic stage [9,17,18]. To be more proactive (to act and not react), studies must be conducted to investigate the predictive value of RDW and its association with the prediabetic stage. Discovering diabetes at an early stage, i.e., prediabetes, is crucial because it provides healthcare providers with more time to intervene via lifestyle modifications; as a consequence, this saves lives and resources [31]. Unfortunately, for untreated individuals with prediabetes, 37% may develop diabetes in four years [31]. Therefore, the aim of the current study was to investigate the association between RDW and prediabetes in adults in River Nile State, Northern Sudan.

## 2. Materials and Methods

### 2.1. Study Area

A case–control study was conducted in River Nile State, Northern Sudan. River Nile State is 1 of the total 18 states of Sudan. River Nile State is adjacent to Khartoum State. According to the last (2008) census, River Nile State has a total population of 1,120,441 [32]. River Nile State has seven localities (according to the Sudan government, it is the locality with the lowest administrative units). Initially, one locality (Almatamah) was selected via simple random sampling. Then, from the Almatamah Locality, three villages were randomly selected.

### 2.2. Participants and Sample Size Selection

All Sudanese adults > 18 years of age from the selected households who agreed to participate in the study were approached by the study research team. The population in each village was taken from the local government authority, and an appropriate sample size was chosen from each village based on the population density of each village. In the case where the household was not inhabited, the neighboring household was chosen so as to obtain the desired sample size (107 for each group).

### 2.3. Data Collection

In the present study, the Strengthening the Reporting of Observational Studies in Epidemiology (STROBE) guidelines were strictly followed [33]. A questionnaire was used to collect the data. Initially, the questionnaire was tested among a small group (10 participants), and all necessary corrections were carried out before data collection. Three trained medical officers interviewed (face-to-face interview) the participants during the study period from May to June 2022. The medical officers explained all necessary information regarding the study (i.e., the aim of the study and ethical issues, including their right to refuse participation at any time without giving any reason). After obtaining signed informed consent forms from the approached participants, the tested questionnaire that included sociodemographic characteristics—such as age in years, sex (male or female), marital status (married or unmarried), education level (<secondary level or secondary level and above), and occupation (employed or not employed)—clinical characteristics—such as family history of DM (yes or no), cigarette smoking (never or current/former), and alcohol consumption (never or current/former)—and anthropometric measurements—including weight and height—were obtained from all participants.

The participants’ weights and heights were measured following the standard procedures, and body mass index was computed from their values using the following equation: weight (kg)/height (m^2^) [34].

After all safety measurements were taken under aseptic conditions from each participant, five milliliters of venous blood sample was collected from each participant in a tube with EDTA for CBC, including RDW and HbA1c analyses.

### 2.4. Definition of the Cases and Controls

In the current study, the cases comprised prediabetic adults (with no known DM) with HbA1c levels ranging from 5.7% to 6.4%. The controls were adults with normal HbA1c levels (<5.7%). Those with known DM or HbAIc levels of ≥6.5%, ages of <18 years, thyroid problems, renal diseases, and pregnancy were excluded from both the case and control groups.

### 2.5. Anthropometric Measurements

Both the weight and height of participants were measured using standard procedures. The participants’ weights were measured in kilograms using standard procedures (well-calibrated scales that were adjusted to zero before each measurement). The participants stood with minimal movement and with arms at their sides. Moreover, shoes and excess clothing were removed. Height was measured in centimeters (later converted into meters to calculate the body mass index) while the participant stood straight with their back against the wall and feet together.

### 2.6. Processing of Blood Samples

The 5 mL blood samples collected under aseptic conditions were transferred to our laboratory for the measurement of CBC, including RDW and HbA1c levels. HbA1c levels were measured via an Ichroma machine in accordance with the manufacturer’s instructions (Republic of Korea); the details of the procedure were previously described in our published work [23]. An automated hematology analyzer was used to measure RDW separately from the CBC, as previously described in our published work [35].

### 2.7. Sample Size Calculation

Open epiinfo software was used to calculate the sample’s size [36]. Therefore, a sample size of 107 participants in each arm of the study was calculated as a ratio of 1:1 between the cases (prediabetes) and controls (healthy participants without DM or prediabetes). The authors assumed a difference of 0.5 in the mean RDW between the prediabetic patients and healthy controls (13.0% vs. 12.5%). Our assumption was dependent on the mean (SD) RDWs of prediabetic patients and healthy controls from a previous study in Turkey [18]. Finally, the sample size (107 participants in each arm) was calculated to detect a difference of 5% at *α* = 0.05 with a power of 80%.

### 2.8. Statistics

The Statistical Package for the Social Sciences for Windows, version 22.0 (IBM, New York, NY, USA), was used to analyze the data. The proportions (categorized data) were expressed as frequencies (%). After evaluation via the Shapiro–Wilk test, all continuous data were found to be not normally distributed, and they were expressed as a median (interquartile range (IQR)). While the Mann–Whitney *U* test was used to compare non-normal distribution data, the chi-square test was used to compare proportions between the two groups (cases and controls). A univariate analysis was performed with prediabetes as the dependent variable and other variables—such as age, sex, education, occupation, cigarette smoking, alcohol consumption, body mass index, and RDW—as independent variables. Thereafter, variables with *p* values of <0.05 were entered in the multivariable regression model to adjust for covariates. Moreover, Spearman correlation analyses were performed to examine the correlation between RDW and HbA1c levels. Adjusted odds ratios (AORs) and 95% confidence intervals (CIs) were calculated as necessary. In this study, a two-sided *p* value of <0.05 was considered statistically significant.

## 3. Results

In this case–control study (107 for each group), sex, educational level, occupational level, marital status, body mass index, cigarette smoking, alcohol consumption, and family history of DM were not associated with prediabetes (*p* value > 0.05) (Table 1).

**Figure 1:**
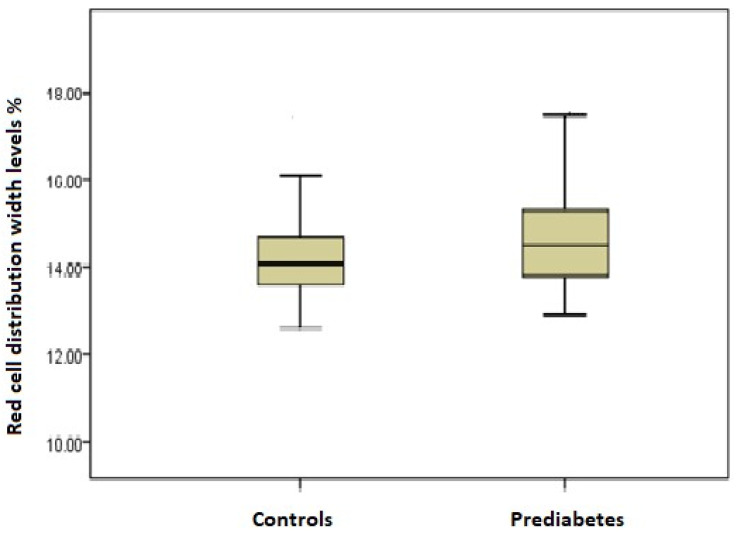
Comparison of red blood cell distribution width between prediabetic patients and controls.

The median (IQR) RDW was significantly higher in the prediabetic patients than in the controls (14.5% [13.8–15.3%] vs. 14.1% [13.6–14.7%], *p* = 0.003; Figure 1).

In the multivariate-adjusted model, age (AOR, 1.02; 95% CI, 1.01–1.04) and RDW (AOR, 1.24; 95% CI, 1.01–1.53) were associated with prediabetes (Table 2). A positive correlation was found between RDW and HbA1c levels (*r* = 0.19, *p* value = 0.006).

## 4. Discussion

As the main finding of the present study, RDW was significantly associated with prediabetes. Several previous studies have reported similar results [9,11,17,18]. In Turkey, a recent study that included 155 patients (T2DM group, n = 45; prediabetes group, n = 60; and control group, n = 50) showed that prediabetes and T2DM were associated with increased RDW [18]. In Korea, a study that included 1470 patients with normoglycemia, 1124 patients with prediabetes, and 396 patients with T2DM concluded that RDW had an independent positive association with pancreatic beta-cell function and insulin resistance in patients with prediabetes and T2DM [9].

In China, a cohort study of 16,971 Chinese adults whose HbA1c levels were measured during annual follow-ups between 2014 and 2019 reported a significant positive association between RDW and an increased incidence of developing DM [11].

On the other hand, some studies have shown no significant association between RDW and DM [15,16]. Our recently published study—which included 253 Sudanese women of early gestational age who completed follow-up—showed no significant difference in hematological parameters, including RDW, between Sudanese women with and without gestational DM [15]. In Iraq, a case–control study that included 30 patients with an established diagnosis of T2DM, 30 patients with T1DM, and 30 apparently healthy controls revealed that RDW had no significant role in predicting poor glycemic control in patients with T1DM or T2DM [16].

The present study shows a positive correlation between RDW and HbA1c levels (*r* = 0.19, *p* = 0.006). Previous studies have shown similar results [17,37]. For example, Tsilingiris et al. reported a significant positive correlation between RDW and HbA1c levels in participants without DM (*r* = 0.315, *p* = 0.001) [17]. Moreover, Bhutto et al. reported a significant positive association between RDW and HbA1c levels in 119 patients with T2DM (*r* = 0.19, *p* = 0.035) [37]. Bao et al. documented that RDW had a stronger association with HbA1c levels than with glucose levels [38]. In their prospective cohort study that included 7795 adults without prediabetes or diabetes who were followed up for a mean of 2.90 years, they concluded that increased RDW is independently related to future increases in HbA1c levels, but not with respect to glucose levels [38]. Furthermore, Bao et al. [38] reported that increased RDW is independently related to future HbA1c elevation, but not with respect to glucose elevation (fasting and postprandial). Such variations between HbA1c, glucose, and RDW need to be taken into consideration when diagnosing DM.

Studies that compared RDW between participants with and without established diabetes have shown contradictory results: that is, a positive association [26,39] or no association between RDW and DM [15,16]. The existing reviewed data indicate a positive association between increased RDW and prediabetes [9,11,17,18]. This could indicate the influence of HbA1c levels on RDW during its early increase, i.e., from 5.7% to 6.4%. This supports the use of RDW as an early biomarker of DM, similarly to our study. However, a lack of association between RDW and established DM (i.e., the presence of contradictory data) necessitates further research about the triggering mechanisms of the influence of HbA1c on RDW and vice versa. Nevertheless, this positive association between prediabetes (HbA1c between 5.7% and 6.4%) and RDW is a promising diabetes predictor tool.

Our results should be cautiously compared with the contradictory findings of previous studies for many reasons. First, our results were obtained from a community-based study (only rural areas), whereas some contradicting results were obtained from facility-based studies. The lack of association between RDW and HbA1c levels in facility-based studies compared to community-based ones, as is the case for our current study, could be attributed to the other healthcare profiles of included participants from facilities; i.e., participants from facilities might have received treatment for certain diseases, lowering their RDW as a consequence. For example, a study conducted by Hu [13] showed that RDW is a promising indicator for estimating the activity of autoimmune diseases, and RDW decreases significantly in treated multiple sclerosis patients [13]. On the other hand, several factors are reported to influence HbA1c levels, such as acute and chronic blood loss; hemolytic anemia; splenomegaly; and iron, folate, and vitamin B-12 deficiencies [40]. This means that being apparently healthy (the controls) does not indicate real health. It is worth mentioning that our recent community-based cross-sectional study showed differences in HbA1c levels with respect to ethnicity and age [41]. Therefore, factors that influence RDW and HbA1c need to be taken into consideration as well. Second, our studied cases only had prediabetes, whereas other studies included patients with known DM. Finally, the difference in the adopted exclusion criteria between the studies might have influenced their discrepant results. For example, the existence of comorbidity and multimorbidity in the same individual might influence the RDW as several diseases can increase RDW, such as autoimmune diseases—including rheumatoid arthritis, systemic lupus erythematosus, inflammatory bowel disease, and inflammatory myopathy [13]—and cardiovascular diseases—including heart failure, stroke, acute coronary syndrome, peripheral artery disease, and hypertension [10].

The mechanisms by which RDW influences blood glucose/HbA1c levels and vice versa are unknown. It is difficult to attribute the present association between increased RDW and prediabetes to coincidence or a real pathophysiological condition. Further studies that investigate the underlying mechanisms between RDW and blood glucose/HbA1c levels are highly recommended. However, various explanations have been reported by researchers, such as the inflammatory process of RDW [42,43] and the influences of hyperglycemia on the lifespan, structure, and function of RBCs [11,44]. Chronic hyperglycemia and hypoinsulinemia are among the components that induce ongoing inflammation, and they play a key role in vascular endothelial growth factor (VEGF) and can lead to diabetic retinopathy [45]. Roque et al. in their case–control study of adult patients (age 18 years and above)—with a total sample size of 262 patients (131 cases with proliferative diabetic retinopathy and 131 controls without proliferative diabetic retinopathy)—concluded that increased RDW values were related to proliferative diabetic retinopathy, suggesting the possible application of RDW as an accessible predictive biomarker of disease progression in patients with diabetic retinopathy [46]. A recent systematic review conducted by Kirthi et al. included 24 studies (8759 participants with prediabetes), and it revealed that the prevalence of diabetic retinopathy ranged from 0.3% to 14.1% [47]. Ma et al. in their a 5-year retrospective case–control study reported that increased RDW values were significantly elevated in diabetic retinopathy patients, and increased RDW was associated with an increased incidence of diabetic retinopathy in patients with DM [27]. Moreover, Ananthaseshan et al. hypothesized that RDW directly affects intravascular hemodynamic interactions between circulating blood cells and vessel walls by inducing local changes that can lead to atherothrombosis [43]. Recently, Poznyak et al. established pathophysiological features that link atherosclerosis to DM via chronic inflammation processes, such as oxidative stress, altered protein kinase signaling, and the role of certain miRNA and epigenetic modifications [48]. Therefore, increased RDW is a novel biomarker for predicting the incidences and prognoses of many diseases, including DM [9,10,12,49,50,51,52,53], especially at the prediabetes stage [9,11,17,18].

As mentioned above, the present study was mainly conducted to assess the association between RDW and prediabetes. In addition to RDW, from the other possible factors that could be associated with prediabetes, only older age was found to be associated with prediabetes. RDW correlated with older age (*r* = 0.150, *p* = 0.029). This result is similar to other studies [8,54]. For instance, in Uganda, Ampeire et al. [8] conducted a cross-sectional survey that included 370 participants aged between 18 and 70 years and revealed a prevalence of 9.19% relative to prediabetes that ranged from 6.23% to 12.14%; the main factors associated with prediabetes were older age, moderate-intensity work, and a high body mass index. Likewise, a study by Xiong et al. included 809 T2DM cases and revealed that older patients with DM had significantly higher RDW levels than younger patients; a high level of RDW in T2DM patients indicates higher risks and a poor prognosis for diabetic nephropathy [54]. Moreover, our previous study in Eastern Sudan including 600 adults showed that older age was among the factors associated with DM [23]. Our current study did not show a positive association between sex, education, body mass index, and alcohol consumption. In contrast, other studies found a positive association [7,8]. Such conflicting results between the present study and other studies could be explained by differences in culture, such as alcohol consumption in different countries. This variation should encourage researchers in each country to obtain their own data and to not completely rely on other countries’ results; in this manner, interventions for combating DM can be precise.

To the best of the authors’ knowledge, this is the first study of its kind that assessed the association between RDW and prediabetes in adults in Sudan. Predicting DM at early stages (i.e., prediabetes) is a good preventive approach for combating DM and its complications, especially in limited-resource settings such as Sudan. Hence, our results showed a significant association between RDW and prediabetes, and we encourage healthcare providers to think about prediabetes/DM whenever there is high RDW; i.e., this study’s results could help healthcare providers, especially in rural and remote areas, with respect to more careful identification, early prevention, intervention, and management in preventing prediabetes/DM and its complications. Therefore, RDW can be used as a predictor of HBA1c levels, and it can be used when considering individuals with high RDW as belonging to high-risk groups of DM and as candidates for further investigations—such as fasting blood glucose and/or HbA1c—in order to rule out prediabetes/DM. Given this, RDW is a routine and inexpensive investigation that can be measured separately to other CBC parameters. In spite of all these added values, our study has some limitations that should be acknowledged and overcome in future studies. First, the present study assessed the association between RDW and HbA1c levels only at one time point. A longitudinal study will provide more clarification regarding such an association between RDW and HbA1c. Second, we only used one indicator (i.e., the RDW-CV value) to characterize the RDW, and this is not the only way to describe the heterogeneity of a cell population, as RDW-SD can be used as well; therefore, there might be a difference between the behavior of these two indicators, i.e., RDW-CV and RDW-SD. Hence, these indicators complement each other, and using them together to identify erythrocyte size heterogeneity is advisable [55]. Third, in this study, we did not collect nutritional information, as a recent study found a direct inverse association between healthy RDW and the omega-3 index in adults of both sexes [19]. Moreover, the current study did not collect information about diets and lifestyles, as other studies have found a positive association between RDW and dietary patterns [19]; lifestyles, including physical activity [21]; and lipid profiles [11]. Finally, this study did not investigate C-reactive protein (CRP) levels, even though a combined effect of CRP and RDW on health prediction has been reported [56]. Conducting another study that takes these raised limitations into account could be of added value with respect to obtaining in-depth information regarding the association between RDW and HbA1c levels.

## 5. Conclusions

A significant association was found between RDW and prediabetes. Therefore, our study supports the use of RDW as a predictor of DM. Further studies that investigate the underlying mechanisms between RDW and blood glucose/HbA1c levels are highly recommended.

## Figures and Tables

**Table 1:** Univariate analysis of sociodemographic and clinical variables in prediabetic patients and controls in Northern Sudan.

Variable		Prediabetic Participants (n = 107)	Controls (n = 107)	Odds Ratio (95% Confidence Interval)	*p* Value
		Range (interquartile range)			
Age (years)		45.0 (35.0–55.0)	37.0 (28.0–51.0)	1.02 (1.01–1.04	0.014
Body mass index (kg/m^2^)		26.6 (22.7–30.5)	25.4 (20.9–30.3)	1.02 (0.97–1.06)	0.478
Red blood cell distribution width (%)		14.5 (13.8–15.3)	14.1 (13.6–14.7	1.25 (1.02–1.54)	0.035
		Frequency (proportion)			
Sex	Male	49 (45.8)	61 (57.0)	Reference	0.101
	Female	58 (54.2)	46 (43.0)	1.57 (0.92–2.69)	
Educational status	<Secondary	74 (69.2)	69 (64.50)	Reference	0.468
	≥Secondary	33 (30.8)	38 (35.5)	1.24 (0.70–2.18)	
Occupational status	Employed	53 (49.5)	53 (49.5)	References	1.000
	Unemployed	54 (50.5)	53 (50.5)	1.00 (0.59–1.71)	
Marital status	Married	27 (25.2)	31 (29.0)	Reference	0.539
	Unmarried	80 (74.8)	76 (71.0)	1.21 (0.66–2.21)	
Family history of diabetes	No	36 (33.6)	28 (26.2)	Reference	0.237
	Yes	71 (66.4)	79 (73.8)	1.45 (0.78–2.69)	
Cigarette smoking	Never	83 (77.6)	81 (75.7)	Reference	0.747
	Current/former	24 (22.4)	26 (24.3)	0.90 (0.48–1.70)	
Alcohol consumption	Never	99 (92.5)	91 (85.0)	Reference	0.089
	Current/former	8 (7.5)	16 (15.0)	0.46 (0.19–1.13)	

**Table 2:** Multivariable analysis of the factors associated with prediabetes for comparisons between prediabetic and healthy adults in Northern Sudan.

Variable	Adjusted Odds Ratio (95% Confidence Interval)	*p* Value
Age (years)	1.02 (1.01–1.04)	0.019
Red blood cell distribution width (%)	1.24 (1.01–1.53)	0.046

## Data Availability

The data presented in this study are available upon request from the corresponding author.

## References

[1] Magliano D.J., Boyko E.J. (2021). IDF Diabetes Atlas 2021.

[2] Harding J.L., Pavkov M.E., Magliano D.J., Shaw J.E., Gregg E.W. (2019). Global trends in diabetes complications: A review of current evidence. Diabetologia.

[3] American Diabetes Association (2010). Standards of medical care in diabetes-2010. Diabetes Care.

[4] Camargo M.S., Passos L.C.S., Mistro S., Soares D.A., Kochergin C.N., de Carvalho V.C.H.d.S., Macedo J.C.L., Cortes T.B.A., de Souza A.A., Rumel D. (2021). Improving access to the glycated hemoglobin test in rural communities with Point-of-Care Devices: An application study. Front. Med..

[5] IDF (2022). The More We Know, the Worse the Picture. https://diabetesatlas.org/.

[6] Zand A., Ibrahim K., Patham B. (2018). Prediabetes: Why should we care?. Methodist Debakey Cardiovasc. J..

[7] Lailler G., Fuentes S., Kab S., Piffaretti C., Guion M., Czernichow S., Cosson E., Fosse-Edorh S. (2023). Prevalence and risk factors associated with prediabetes and undiagnosed diabetes in France: The national CONSTANCES cohort. Diabetes Epidemiol. Manag..

[8] Ampeire I.P., Kawugezi P.C., Mulogo E.M. (2023). Prevalence of prediabetes and associated factors among community members in rural Isingiro district. BMC Public Health.

[9] Nah E.H., Cho S., Park H., Kim S., Cho H.I. (2022). Associations of complete blood count parameters with pancreatic beta-cell function and insulin resistance in prediabetes and type 2 diabetes mellitus. J. Clin. Lab. Anal..

[10] Arkew M., Gemechu K., Haile K., Asmerom H. (2022). Red blood cell distribution width as novel biomarker in cardiovascular diseases: A literature review. J. Blood Med..

[11] Wang J., Zhang Y., Wan Y., Fan Z., Xu R. (2020). The relationship between Red blood cell distribution width and incident diabetes in Chinese adults: A cohort study. J. Diabetes Res..

[12] Uzun F., Güner A., Pusuroglu H., Demir A.R., Gündüz S., Gürbak İ., Aslan S., Demirci G., Gültekin Güner E., Arslan E. (2022). Association of red blood cell distribution width, systemic-immune-inflammation index and poor cardiovascular outcomes in patients with newly diagnosed hypertension. Clin. Exp. Hypertens..

[13] Hu Z.-D. (2016). Red blood cell distribution width: A promising index for estimating activity of autoimmune disease. J. Lab. Precis. Med..

[14] Mo M., Huang Z., Huo D., Pan L., Yang Z., Xia N., Liao Y. (2022). Influence of red blood cell distribution width on all-cause death in critical diabetic patients with acute kidney anjury. Diabetes Metab. Syndr. Obes. Targets Ther..

[15] Hassan B., Rayis D.A., Musa I.R., Eltayeb R., ALhabardi N., Adam I. (2021). Blood groups and hematological parameters do not associate with first trimester gestational diabetes mellitus (institutional experience). Ann. Clin. Lab. Sci..

[16] Dibby H.J., Shlash R.F. (2020). The predictive value of red cell distribution (RDW) in patients with type 1 and type 2 diabetes mellitus. Medico-Legal Updat..

[17] Tsilingiris D., Makrilakis K., Barmpagianni A., Dalamaga M., Tentolouris A., Kosta O., Eleftheriadou I., Liatis S. (2021). The glycemic status determines the direction of the relationship between red cell distribution width and HbA1c. J. Diabetes Complicat..

[18] Kavvasoglu B., Akdemir S.N., Kurt M. (2022). A routine but overlooked parameter for impaired glucose control: Red cell distribution width. Eur. Rev. Med. Pharmacol. Sci..

[19] McBurney M.I., Tintle N.L., Harris W.S. (2022). Omega-3 index is directly associated with a healthy red blood cell distribution width. Prostaglandins Leukot. Essent. Fat. Acids.

[20] Aldosari K.H., Ahmad G., Al-Ghamdi S., Alsharif M.H.K., Elamin A.Y., Musthafa M., Abbas M.Y., Alqarni A.A., Alqudeebi S.K., Binsaqer A.A. (2020). The influence and impact of smoking on red blood cell morphology and buccal microflora: A case-control study. J. Clin. Lab. Anal..

[21] Emans M.E., Gaillard C.A.J.M., Pfister R., Tanck M.W., Boekholdt S.M., Wareham N.J., Khaw K.T. (2013). Red cell distribution width is associated with physical inactivity and heart failure, independent of established risk factors, inflammation or iron metabolism; the EPIC—Norfolk study. Int. J. Cardiol..

[22] Omar S.M., Musa I.R., Idrees M.B., Adam I. (2021). Prevalence of depression and associated factors among patients with type 2 diabetes mellitus in eastern Sudan. BMC Psychiatry.

[23] Omar S.M., Musa I.R., ElSouli A., Adam I. (2019). Prevalence, risk factors, and glycaemic control of type 2 diabetes mellitus in eastern Sudan: A community-based study. Ther. Adv. Endocrinol. Metab..

[24] Elrayah-Eliadarous H.A., Östenson C.G., Eltom M., Johansson P., Sparring V., Wahlström R. (2017). Economic and social impact of diabetes mellitus in a low-income country: A case-control study in Sudan. J. Diabetes.

[25] Asmelash D., Asmelash Y. (2019). The burden of undiagnosed diabetes mellitus in adult african population: A systematic review and meta-analysis. J. Diabetes Res..

[26] Arkew M., Asmerom H., Tesfa T., Tsegaye S., Gemechu K., Bete T., Haile K. (2022). Red blood cell parameters and their correlation with glycemic control among type 2 diabetic adult patients in eastern Ethiopia: A comparative cross-sectional study. Diabetes Metab. Syndr. Obes. Targets Ther..

[27] Ma Y., Li S., Zhang A., Ma Y., Wan Y., Han J., Cao W., Xu G. (2021). Association between red blood cell distribution width and diabetic retinopathy: A 5-year retrospective case-control study. J. Ophthalmol..

[28] Wang L., Lv Y. (2022). Construction of a Prediction Model for the Mortality of Elderly Patients with Diabetic Nephropathy. J. Healthc. Eng..

[29] Assulyn T., Khamisy-Farah R., Nseir W., Bashkin A., Farah R. (2020). Neutrophil-to-lymphocyte ratio and red blood cell distribution width as predictors of microalbuminuria in type 2 diabetes. J. Clin. Lab. Anal..

[30] Dai H., Su X., Li H., Zhu L. (2020). Association between red blood cell distribution width and mortality in diabetic ketoacidosis. J. Int. Med. Res..

[31] Tuso P. (2014). Prediabetes and lifestyle modification: Time to prevent a preventable disease. Perm. J..

[32] Sudan Government (2009). 5th Sudan Population and Housing Census—2008. https://catalog.ihsn.org/index.php/catalog/4216/do.

[33] von Elm E., Altman D.G., Egger M., Pocock S.J., Gøtzsche P.C., Vandenbroucke J.P. (2008). The Strengthening the Reporting of Observational Studies in Epidemiology (STROBE) statement: Guidelines for reporting observational studies. J. Clin. Epidemiol..

[34] World Health Organization Obesity: Preventing and Managing the Global Epidemic. Report of a WHO Consultation. https://apps.who.int/iris/handle/10665/42330.

[35] Abdelrahman E.G., Gasim G.I., Musa I.R., Elbashir L.M., Adam I. (2012). Red blood cell distribution width and iron deficiency anemia among pregnant Sudanese women. Diagn. Pathol..

[36] Dean A.G., Sullivan K.M., Soe M.M. (2013). OpenEpi: Open Source Epidemiologic Statistics for Public Health, Version. www.OpenEpi.com.

[37] Bhutto A.R., Abbasi A., Abro A.H. (2019). Correlation of hemoglobin A1c with red cell width distribution and other parameters of red blood cells in type II diabetes mellitus. Cureus.

[38] Bao X., Wan M., Gu Y., Song Y., Zhang Q., Liu L., Meng G., Wu H., Xia Y., Shi H.B. (2017). Red cell distribution width is associated with hemoglobin A1C elevation, but not glucose elevation. J. Diabetes Complicat..

[39] Shirali S., Bahadoram S., Nourbakhsh S.M.K., Bahadoram M., Valipour A.A., Javanmardi F. (2018). Red cell distribution width as a prognostic marker in diabetes; a pilot cross-sectional study. J. Nephropharmacol..

[40] Radin M.S. (2014). Pitfalls in hemoglobin A1c measurement: When results may be misleading. J. Gen. Intern. Med..

[41] Ahmed S.F., Hassan A.A., Eltayeb M.M., Omar S.M., Adam I. (2023). Ethnicity, age, and gender differences in glycated hemoglobin (HbA1c) levels among adults in Northern and Eastern Sudan: A community-based cross-sectional study. Life.

[42] Bilal A., Farooq J.H., Kiani I., Assad S., Ghazanfar H., Ahmed I. (2016). Importance of mean red cell distribution width in hypertensive patients. Cureus.

[43] Ananthaseshan S., Bojakowski K., Sacharczuk M., Poznanski P., Skiba D.S., Prahl Wittberg L., McKenzie J., Szkulmowska A., Berg N., Andziak P. (2022). Red blood cell distribution width is associated with increased interactions of blood cells with vascular wall. Sci. Rep..

[44] Wang Y., Yang P., Yan Z., Liu Z., Ma Q., Zhang Z., Wang Y., Su Y. (2021). The relationship between erythrocytes and diabetes mellitus. J. Diabetes Res..

[45] Gomułka K., Ruta M. (2023). The role of inflammation and therapeutic concepts in diabetic retinopathy—A short review. Int. J. Mol. Sci..

[46] Roque J.C., Quezada G., Saldaña C., Carrillo C., Vargas J.A. (2020). Red blood cell distribution width an inflammatory biomarker related to proliferative diabetic retinopathy. Rev. Fac. Med. Humana.

[47] Kirthi V., Nderitu P., Alam U., Evans J.R., Nevitt S., Malik R.A., Hopkins D., Jackson T.L. (2022). The prevalence of retinopathy in prediabetes: A systematic review. Surv. Ophthalmol..

[48] Poznyak A., Grechko A.V., Poggio P., Myasoedova V.A., Alfieri V., Orekhov A.N. (2020). The diabetes mellitus–atherosclerosis connection: The role of lipid and glucose metabolism and chronic inflammation. Int. J. Mol. Sci..

[49] Sileshi B., Urgessa F., Wordofa M. (2021). A comparative study of hematological parameters between hypertensive and normotensive individuals in Harar, eastern Ethiopia. PLoS ONE.

[50] Vinoj J., Vignesh D. (2022). To evaluate the association between red cell distribution width and validated neurological scores in patients with acute stroke. J. Evol. Med. Dent. Sci..

[51] Diab E.E., Abdallah E.I., Elbasheir M.M. (2020). Red blood cell distribuation (RDW) as a predictive biomarker for patients with myocarial infaction in Sudan. Eur. J. Pharm. Med. Res..

[52] Zinellu A., Mangoni A.A. (2022). The emerging clinical significance of the red cell distribution width as a biomarker in chronic obstructive pulmonary disease: A systematic review. J. Clin. Med..

[53] Erdogan A., Keskin E., Sambel M. (2022). Red blood cell distribution width values in erectile dysfunction. Rev. Int. Androl..

[54] Xiong X.F., Yang Y., Chen X., Zhu X., Hu C., Han Y., Zhao L., Liu F., Sun L. (2017). Red cell distribution width as a significant indicator of medication and prognosis in type 2 diabetic patients. Sci. Rep..

[55] Caporal F.A., Comar S.R. (2013). Evaluation of RDW-CV, RDW-SD, and MATH-1SD for the detection of erythrocyte anisocytosis observed by optical microscopy. J. Bras. Patol. Med. Lab..

[56] Wei X.B., Liu Y.H., He P.C., Zhou Y.L., Tan N., Chen J.Y., Yu D.Q. (2017). Combined efficacy of C-reactive protein and red blood cell distribution width in prognosis of patients with culture-negative infective endocarditis. Oncotarget.

